# Repurposing PDE5-Inhibitors: Sildenafil Drives Arteriogenesis via Localized Regenerative Inflammation

**DOI:** 10.3390/ijms27020985

**Published:** 2026-01-19

**Authors:** Katharina Elbs, Lisa Bobrowski, Christoph Arnholdt, Matthias Kübler, Philipp Götz, Michael R. Rohrmoser, Daphne Merkus, Manuel Lasch, Elisabeth Deindl

**Affiliations:** 1 Institute of Surgical Research at the Walter-Brendel-Centre of Experimental Medicine, University Hospital, Ludwig-Maximilians-Universität München, 81377 Munich, Germany; katharina.elbs@med.uni-muenchen.de (K.E.); lisa.bobrowski@med.uni-muenchen.de (L.B.); christophjohannes.arnholdt@med.uni-heidelberg.de (C.A.); matthias.kuebler@med.uni-muenchen.de (M.K.); p.goetz@med.uni-muenchen.de (P.G.); michael.rohrmoser@med.uni-muenchen.de (M.R.R.); daphne.merkus@med.uni-muenchen.de (D.M.); manuel.lasch@med.uni-muenchen.de (M.L.); 2 Biomedical Center, Institute of Cardiovascular Physiology and Pathophysiology, Faculty of Medicine, Ludwig-Maximilians-Universität München, 82152 Planegg, Germany; 3 Department of Cardiovascular Diseases, TUM University Hospital German Heart Center, 80636 Munich, Germany; 4 Department of Ophthalmology, University of Heidelberg, 69120 Heidelberg, Germany; 5 Deutsches Zentrum Immuntherapie (DZI), Comprehensive Cancer Center Erlangen-EMN (CCC ER-EMN), Friedrich-Alexander-Universität Erlangen-Nürnberg (FAU), 91054 Erlangen, Germany; 6 Department of Oral- and Cranio-Maxillofacial Surgery, Friedrich-Alexander-Universität Erlangen-Nürnberg (FAU), 91054 Erlangen, Germany; 7 Department of Otorhinolaryngology, Head and Neck Surgery, Heidelberg University Hospital, 69120 Heidelberg, Germany; 8 Department of Experimental Cardiology, Erasmus University Medical Center, 3015 GD Rotterdam, The Netherlands; 9 Center for Cardiovascular Research (DZHK), Munich Heart Alliance (MHA), Partner Site Munich, 81377 Munich, Germany; 10 Department of Otorhinolaryngology, Head and Neck Surgery, University Hospital Munich, Ludwig-Maximilians-Universität München, 81377 Munich, Germany

**Keywords:** PDE5, PDE5-inhibitor, Sildenafil, platelet activation, collateral artery growth, cardiovascular occlusive disease, peripheral artery disease, regenerative inflammation, natural bypass growth

## Abstract

Arteriogenesis, the growth of pre-existing arterioles into functional collateral arteries, represents a key adaptive response to severe arterial stenosis. This process is driven by hemodynamic forces and a tightly coordinated inflammatory cascade. Here, we investigated the effects of pharmacological stimulation of the nitric oxide-cyclic guanosine monophosphate (NO-cGMP) signaling pathway using the phosphodiesterase-5 (PDE5) inhibitor Sildenafil on collateral vessel growth in a murine model of femoral artery ligation (FAL). Flow cytometric analyses revealed that Sildenafil treatment significantly enhanced platelet–leukocyte aggregate formation, a prerequisite for the subsequent initiation of a localized perivascular inflammation. Histological and immunofluorescence analyses further demonstrated a marked increase in mast cell recruitment and degranulation at early time points (days 1 and 3 post-FAL). In addition, Sildenafil promoted perivascular macrophage accumulation on days 3 and 7, with a pronounced shift toward an M2-like pro-regenerative polarization state, ultimately resulting in the enhanced proliferation of vascular cells and the enlargement of collateral diameters. Together, these findings identify Sildenafil as a potent enhancer of arteriogenesis through coordinated immune cell activation, stimulating vascular cell proliferation along with positive collateral outward remodeling. Thus, Sildenafil emerges as a promising therapeutic candidate to promote collateral artery growth in cardiovascular occlusive diseases.

## 1. Introduction

Addressing cardiovascular occlusive diseases, such as coronary artery disease or peripheral artery disease, using non-invasive treatment options represents an emerging objective in modern medicine [1,2]. A particularly promising strategy involves stimulating the growth of natural arterial bypasses. This describes the process of outward remodeling of preexisting collateral arteries in response to a relevant narrowing of a main artery [3,4]. Fostering this endogenous capacity for positive vascular adaptation might be preferable to invasive procedures such as bypass surgery or percutaneous stenting, especially for high-risk patients undergoing invasive procedures or lacking access to specialized medical treatment.

In contrast to ischemia-triggered angiogenesis, that occurs in the musculature distal to the stenosis of the main artery, natural bypass growth, termed arteriogenesis, is initiated by enhanced laminar shear stress in the pre-existing collateral arteries that bypass the site of the stenosis [5,6]. This biomechanical stimulus induces endothelial activation and downstream signaling that orchestrates the outward remodeling process of the natural bypasses. Notably, flow directionality and shear stress patterns critically influence endothelial gene expression and the recruitment of immune cells, which are essential for collateral artery growth, highlighting the complex mechanotransduction pathways driving arteriogenesis [7]. Activated endothelial cells release von Willebrand factor (VWF), which triggers platelet activation, leading to the formation of platelet–leukocyte aggregates (PLA). PLA formation is essential for the recruitment of immune cells, such as mast cells, to the perivascular space of growing collateral arteries and their subsequent activation [8,9]. This process results in perivascular inflammation, characterized by the accumulation of initially inflammatory and later regenerative macrophage subsets. These subsets are crucial for the outward remodeling process and the proliferation of vascular cells, ultimately facilitating arteriogenesis [10,11].

One promising approach to promote arteriogenesis is to stimulate nitric oxide (NO)-signaling. In the past, NO-donor application has been shown to enhance arteriogenesis [12]. However, NO-donors are not feasible for long-term treatment of patients with cardiovascular diseases (CVDs) due to rapid tolerance development [13]. Furthermore, recent studies have demonstrated that alterations in the NO-signaling pathways, which decrease NO signaling, are linked to an enhanced risk for CVDs [14,15,16]. Particularly, the upregulation of phosphodiesterase-5 (PDE5) activity is strongly associated with an increased risk of CVDs [17,18,19,20]. PDE5 degrades cyclic guanosine monophosphate (cGMP) and thereby terminates NO signaling. Inhibiting PDE5 limits the degradation of cGMP, thereby enhancing its bioavailability and stimulating the NO pathway [21]. Consequently, PDE5 inhibition may serve as a potential treatment strategy to promote arteriogenesis, specifically in patients with CVDs.

Sildenafil, a selective PDE5-inhibitor, has been extensively studied for the treatment of erectile dysfunction (ED) [22], pulmonary arterial hypertension (PAH) [23], microvascular diseases [24,25], platelet function [26,27,28], and angiogenesis [29,30]. It is a particularly widely prescribed drug for the treatment of erectile dysfunction [31] and PAH and has been shown to be generally safe in patients with CVDs [23].

However, its effect on the growth of collateral arteries has yet to be evaluated. Therefore, we aimed to investigate the impact of Sildenafil on arteriogenesis in our well-established murine hindlimb model [32] in this study.

## 2. Results

### 2.1. Sildenafil Boosts Perfusion Recovery After Femoral Artery Ligation

Arteriogenesis was induced through minimally invasive unilateral femoral artery ligation (FAL) with a sham operation on the contralateral leg serving as an internal control. Sildenafil treatment began 24 h prior to the surgical intervention to ensure that therapeutic drug levels were already present at the time arteriogenesis was triggered.

**Figure 1:**
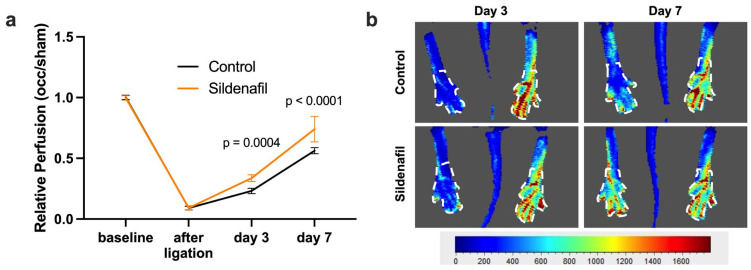
Perfusion Recovery after Femoral Artery Ligation (FAL) is Promoted by Sildenafil. (**a**) The graph depicts the relative perfusion (calculated as the ratio of blood flow in the limb distal to the occlusion and the contralateral sham side) after induction of arteriogenesis by FAL in control (black line) or Sildenafil-treated (orange line) mice directly prior to (baseline) and immediately after FAL and on days 3 and 7 after FAL as assessed by laser Doppler imaging (LDI). Statistical analysis was performed using a two-way ANOVA followed by a Bonferroni post hoc correction (*n* = 6 animals per group). (**b**) Representative flux images from LDI show the murine hindlimb on days 3 and 7 after FAL in control and Sildenafil-treated animals. The color scale reflects relative perfusion levels ranging from low (blue) to high (red). White dashed outlines indicate the regions of interest that were selected for quantitative analysis.

Laser Doppler imaging (LDI) was used to assess initial limb perfusion as well as the restoration of blood flow over time after FAL. As expected, FAL resulted in an immediate reduction in the perfusion in the limb on the occluded side, reflected by a drop in the perfusion ratio between the occluded and contralateral sham side. Over the following days, both the Sildenafil-treated group and the control group exhibited a gradual improvement in relative perfusion, with measurable perfusion recovery occurring by days 3 and 7 post-FAL. Notably, mice receiving Sildenafil displayed a significantly greater restoration of relative perfusion at both time points compared with the controls (Figure 1).

### 2.2. PDE5 Inhibition Promotes Vascular Cell Proliferation upon FAL

**Figure 2:**
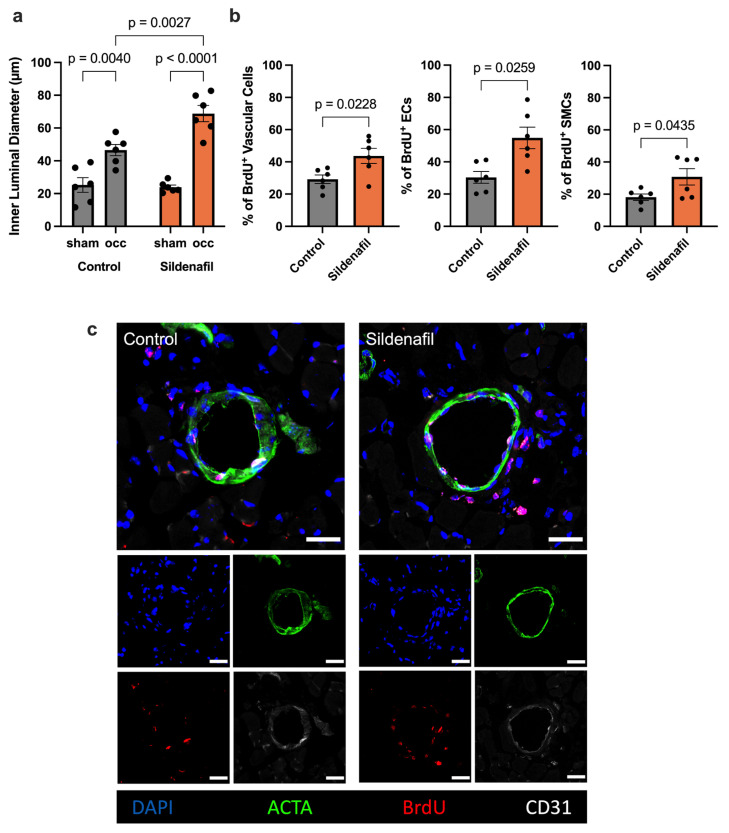
Treatment with Sildenafil Increases Inner Collateral Artery Diameters and Vascular Cell Proliferation. (**a**) The graph demonstrates the inner luminal diameter of the growing (occ) compared to resting (sham) collateral arteries in Sildenafil treated or control animals 7 days after femoral artery ligation (FAL). Statistical analysis was conducted with Two-Way ANOVA with Bonferroni-Correction (*n* = 6 animals per group). (**b**) The bar graphs illustrate the proportion of proliferating total vascular cells (left), endothelial cells (ECs) or smooth muscle cells (SMCs) relative to all vascular cells, ECs or SMCs, respectively, as indicated by bromodeoxyuridine incorporation (BrdU^+^) in Sildenafil treated or control animals on day 7 after FAL. Statistical comparisons between groups were conducted using an unpaired *t*-test (*n* = 6 animals per group). (**c**) Representative immunofluorescence images of collateral arteries obtained from Sildenafil-treated or control mice at day 7 post-FAL are shown. Cell nuclei are stained with DAPI (blue), SMCs are visualized by anti-alpha smooth muscle actin (ACTA2, green), ECs are marked by CD31 (white), and proliferating cells are identified by BrdU incorporation (red). Areas where BrdU and CD31 signals overlap are depicted in pink. Scale bar represents 25 μm.

To validate whether our finding of increased perfusion recovery after FAL in Sildenafil-treated mice compared to controls reflected true arteriogenesis and not simply vasodilation, we performed immunofluorescence staining of the collateral arteries using bromodeoxyuridine (BrdU) as a marker for proliferation, CD31 as a marker for endothelial cells, and alpha smooth muscle actin (ACTA) to identify vascular smooth muscle cells. This analysis demonstrated larger inner luminal diameters of growing collateral arteries after femoral artery ligation in comparison to the respective resting collaterals on the sham side. Furthermore, Sildenafil-treated mice exhibited a significant increase in the luminal diameter of growing collateral arteries compared to the growing collateral arteries of control mice (Figure 2a,c). Additionally, treatment with Sildenafil significantly increased total vascular cell proliferation on day 7 after FAL compared to controls. Specifically, Sildenafil treatment boosted both vascular endothelial and smooth muscle cell proliferation after induction of arteriogenesis by FAL when compared to the controls (Figure 2b,c).

### 2.3. Sildenafil Boosts Platelet–Leukocyte Interaction After Induction of Arteriogenesis

**Figure 3:**
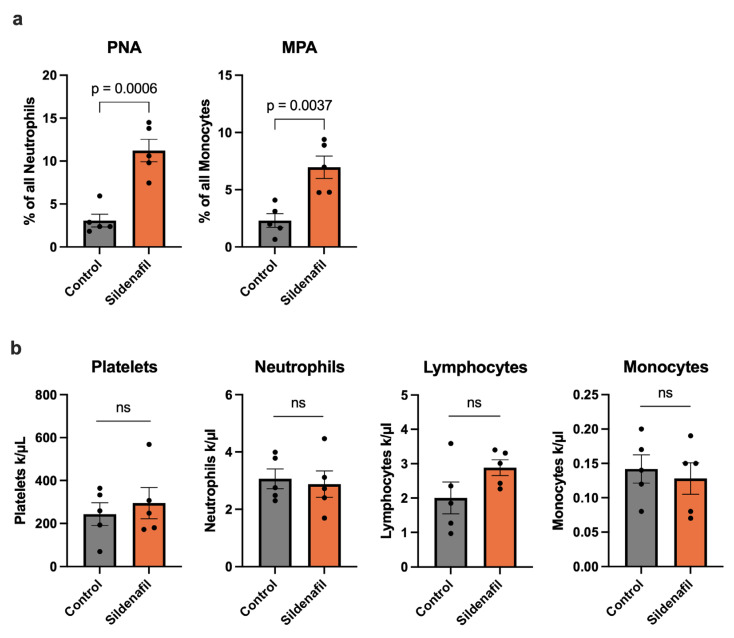
Sildenafil Triggers Circulating Platelet–Neutrophil Aggregate (PNA) and Monocyte–Platelet Aggregate (MPA) Formation During Arteriogenesis. (**a**) The bar graphs demonstrate the percentage of PNA of all neutrophils and MPA of all monocytes in Sildenafil-treated or control mice 24 h after femoral artery ligation (FAL) detected by flow cytometry. (**b**) The bar graphs depict the numbers of platelets, neutrophils, lymphocytes, and monocytes as evaluated by differential blood count in Sildenafil-treated or control animals 24 h after FAL. (**a**,**b**) Statistical analysis was performed using an unpaired *t*-test (*n* = 5 animals per group). ns = not significant.

A rise in shear stress within preexisting collateral vessels triggers a series of early cellular events, among which platelet activation and their association with circulating leukocytes play a central role [8,9]. To assess these interactions, we performed flow cytometric profiling of whole blood obtained 24 h after FAL, a time point corresponding to the initial phase of arteriogenesis. In mice receiving Sildenafil, we observed a marked elevation in the proportion of neutrophils forming complexes with platelets, identified as CD41^+^/GR-1^+^ platelet–neutrophil aggregates (PNA). A similar increase was detected for monocytes engaging with platelets, quantified as CD41^+^/CD115^+^ monocyte–platelet aggregates (MPA) in animals that received Sildenafil treatment compared to controls (Figure 3a). To exclude the possibility that these changes were due to alterations in circulating blood cell populations, a differential blood count of whole blood samples harvested 24 h after FAL was performed. Here, Sildenafil did not influence the platelet, neutrophil, lymphocyte, or monocyte counts, respectively, compared to the control mice (Figure 3b).

### 2.4. Sildenafil Increases Mast Cell Recruitment and Degranulation

**Figure 4:**
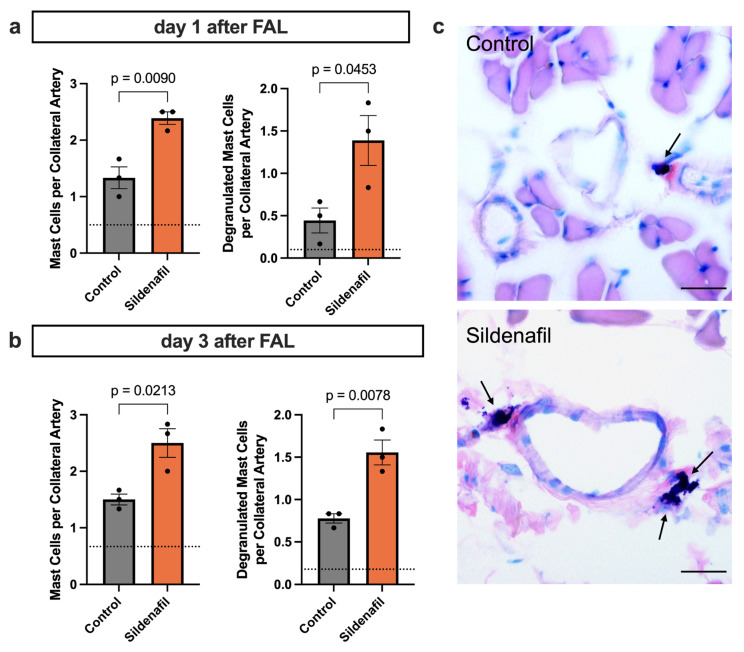
PDE5 Inhibition Promotes Perivascular Mast Cell Recruitment and Degranulation During Arteriogenesis. (**a**,**b**) The bar charts present the quantification of perivascular mast cells, expressed as mast cells per collateral artery (left panels), as well as the number of degranulated mast cells per collateral artery (right panels), in mice treated with Sildenafil compared with controls. These analyses were performed on Giemsa-stained muscle sections collected either (**a**) 1 or (**b**) 3 days after femoral artery ligation (FAL), counting only mast cells in direct proximity to the growing collateral arteries. The dotted horizontal line indicates the mean mast cell count obtained from the contralateral sham-operated side. Statistical evaluation was carried out using an unpaired *t*-test (*n* = 3 mice per group). (**c**) Representative Giemsa-stained images highlighting perivascular mast cells (arrows) 3 days after FAL. Scale bar = 25 µm.

PLA formation is associated with the recruitment and activation of mast cells during arteriogenesis [9]. To investigate whether pharmacological stimulation of the NO-cGMP pathway affects mast cell dynamics during natural bypass growth, we analyzed collateral artery cross-sections from animals treated with Sildenafil or control mice following FAL. Giemsa staining was used to quantify mast cell recruitment and degranulation in the perivascular region of growing collaterals at day 1 and day 3 after FAL. Sildenafil treatment resulted in a marked increase in the number of perivascular mast cells as early as 24 h after FAL compared to the control group (Figure 4a). Moreover, the proportion of degranulated mast cells was significantly higher in the Sildenafil group, indicating enhanced activation of these cells during the early phase of collateral growth (Figure 4a). By day 3 post-FAL, Sildenafil-treated animals continued to exhibit elevated mast cell accumulation in the perivascular space compared to the control group, along with persistently increased levels of degranulating mast cells (Figure 4b,c).

### 2.5. PDE5 Inhibition Drives Perivascular Regenerative Inflammation

**Figure 5:**
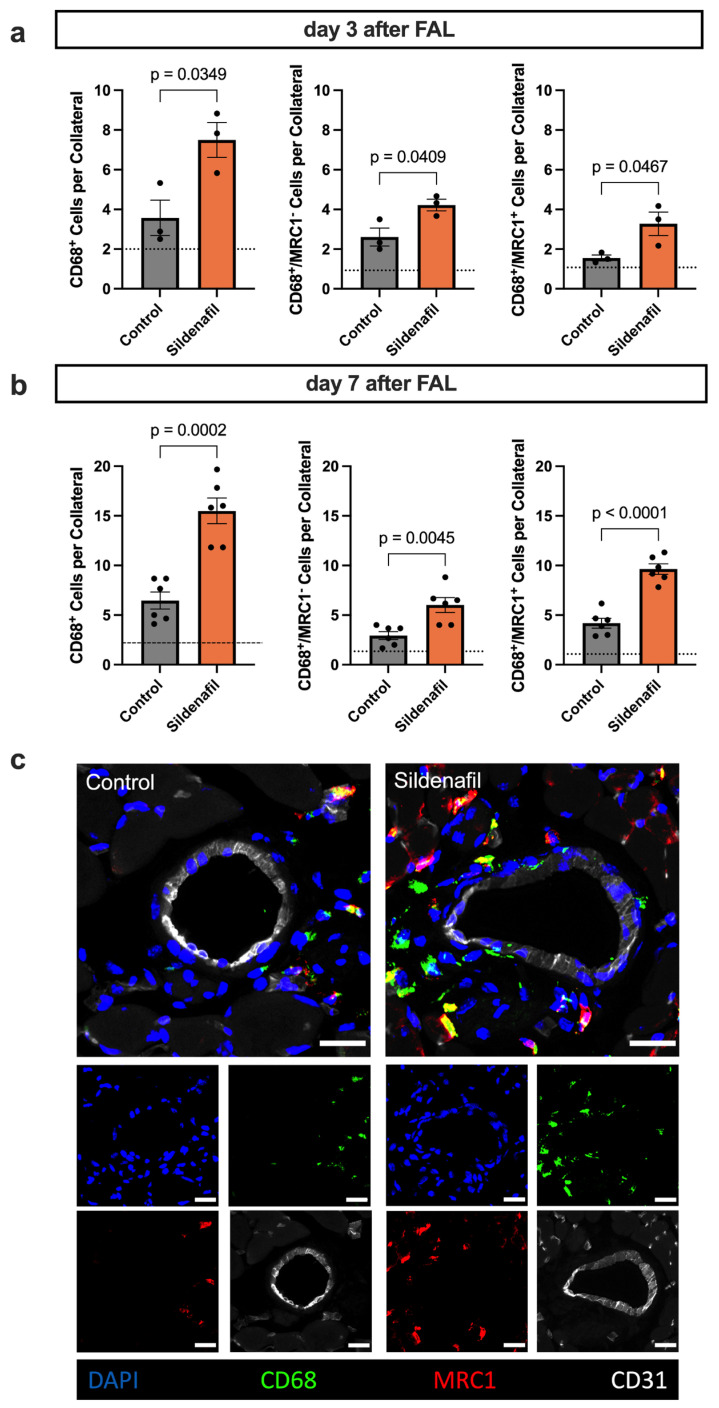
Sildenafil Triggers Macrophage Recruitment and Shifts Macrophage Polarization. (**a**,**b**) The bar graphs demonstrate the total number of macrophages (CD68^+^) per collateral artery (left), the number of M1-like polarized macrophages (CD68^+^/mannose receptor C type 1 (MRC)^−^) per collateral artery (middle) and the number of M2-like polarized macrophages (CD68^+^/MRC1^+^) per collateral artery on (**a**) day 3 or (**b**) day 7 after induction of arteriogenesis. The dotted line represents the average value of the sham side. Statistical analysis was performed by an unpaired *t*-test ((**a**) *n* = 3 animals per group, (**b**) *n* = 6 animals per group). For quantitative evaluation, only the macrophages in the perivascular region up to a distance that excluded the surrounding skeletal muscle fibers were included. (**c**) Representative immunofluorescence staining of collateral arteries (CD31^+^ marking endothelial cells in white) and surrounding macrophages (CD68^+^ in green) with M1-like (MRC1^−^) or M2-like (MRC1^+^ in red) polarization. The larger images show the merge of the smaller single-channel images. Scale bar = 25 µm.

Mast cell activation and the release of several cytokines result in the recruitment of immune cells to the perivascular space of the growing collateral arteries [9]. To determine how Sildenafil influences the perivascular inflammation during collateral vessel growth, we performed immunofluorescence staining on adductor muscle sections collected at days 3 and 7 after induction of arteriogenesis by FAL. This approach enabled us to characterize the temporal pattern of macrophage infiltration and the direction of polarization (M1-like pro-inflammatory or M2-like pro-regenerative) in response to pharmacological activation of the NO-cGMP pathway by Sildenafil. On day 3 after FAL, Sildenafil administration led to a significantly increased accumulation of macrophages (CD68^+^) in the perivascular region of growing collateral arteries compared to control animals (Figure 5a). At this early stage, both M1-like (CD68^+^/mannose receptor C type 1 (MRC1)^−^) and M2-like (CD68^+^/MRC1^+^) polarized macrophage subsets were elevated, showing a generally enhanced inflammatory response. By day 7 after FAL, the overall number of perivascular macrophages remained significantly higher in the Sildenafil group compared to the control group (Figure 5b,c). Importantly, while both subsets continued to increase, the expansion of M2-like polarized macrophages was more pronounced, suggesting that Sildenafil fosters a phenotypic shift toward an anti-inflammatory and tissue-remodeling macrophage profile during later phases of arteriogenesis.

## 3. Discussion

In this study, we demonstrate that Sildenafil markedly enhances collateral artery growth following femoral artery ligation, highlighting the therapeutic potential of stimulating the NO-cGMP pathway in promoting arteriogenesis. Beyond its known vasodilatory properties, Sildenafil positively modulated both early and late inflammatory responses to the occluded main artery, ultimately leading to the promotion of collateral artery cell proliferation, resulting in the increase in collateral artery diameters.

PDE5 inhibition is mostly known for its acute vasodilator effects on the coronary, systemic, and pulmonary vasculature [33,34], as well as its beneficial effects on pulmonary vascular remodeling in PAH [35]. Here, we study the effect of Sildenafil on arteriogenesis in mice undergoing unilateral femoral artery ligation, a standard animal model for evaluating arteriogenesis [36]. Our findings reveal a significant improvement in perfusion recovery compared to controls, which is consistent with previous work that has highlighted the positive impact of Sildenafil on blood-flow redistribution after carotid artery occlusion, an effect that is likely mediated by improved collateral circulation [37]. Furthermore, Sildenafil was shown to induce signaling pathways involving the phosphoinositide 3-kinase, protein kinase B, and the endothelial NO synthase, contributing to tissue perfusion after femoral artery ligation [38] and to ameliorate angiogenesis, further supporting a broader therapeutic application for PDE5 inhibition in advancing vascular remodeling processes [29].

Since Sildenafil is well-known as a vasodilating agent [39,40], it is important to differentiate between the actual growth of collateral arteries and transient vasodilation, which can also temporarily increase blood flow to the peripheral hindlimbs following FAL. Our immunofluorescence stains clearly show the presence of proliferating vascular cells in the collateral arteries. The number of proliferating vascular endothelial and smooth muscle cells, accompanied by enhanced inner collateral diameters, was significantly higher in Sildenafil-treated animals compared to the controls. This pro-proliferative effect is highly context dependent, as previous studies have reported both anti-proliferative [41,42] and pro-proliferative [43] effects of Sildenafil on various vascular endothelial and smooth muscle cells in vitro. However, our data clearly demonstrate that Sildenafil promotes vascular cell proliferation in vivo under arteriogenic conditions.

It is important to note that arteriogenesis is primarily driven by increased shear stress [5,44], which is known to increase NO and cGMP and to trigger endothelial cell activation and the subsequent remodeling of pre-existing arterioles into functional collateral arteries. Upon activation, endothelial cells release VWF, which activates platelets, resulting in their interaction with leukocytes [8,9,45]. In particular, the formation of PNA and MPA has been reported following the induction of arteriogenesis [9,46]. In the present study, we demonstrate that Sildenafil treatment significantly upregulates the formation of PNA and MPA after the induction of arteriogenesis. At the same time, the absolute numbers of circulating platelets, neutrophils, monocytes and lymphocytes remained unchanged by Sildenafil treatment when compared to control mice.

The interaction of platelets with leukocytes is mediated by the transient binding of platelet P-selectin with its corresponding receptor on leukocytes [9,47], and requires prior platelet activation and P-selectin surface exposure [48]. While the inhibitory role of cGMP-signaling in platelets in platelet aggregation and thrombus formation is well known [49,50], its role in regulating platelet–leukocyte interactions remains poorly understood. Previous studies demonstrated that PDE5 inhibition increases platelet cGMP levels, underscoring its direct effects on platelets [51,52]. Notably, PDE5 inhibition has been described to exert stimulus-dependent effects on platelet activation: while inhibitory effects on platelet activation have been reported for collagen-, adenosine diphosphate (ADP)- and protease-activated receptor 1 (PAR1)-mediated pathways, only minor effects have been described for glycoprotein IV-mediated platelet activation [26,53]. On the contrary, stimulation of the cGMP-dependent protein kinase G (PKG) pathway has been discussed to selectively stimulate glycoprotein receptor Ib (GPIb)-dependent platelet activation [54], a pathway critically involved in shear-stress dependent platelet activation during arteriogenesis. Thus, the effect of PDE5 inhibition on platelets seems to be multifaceted and highly dependent on the activating stimulus. In the context of arteriogenesis, where GPIb-mediated platelet activation is particularly relevant, enhanced cGMP-signaling may favor P-selectin exposure and thereby promote the subsequent formation of PLA, as observed in the present study.

While the biological function of MPA formation during arteriogenesis remains unclear, platelet activation itself initiates several downstream cellular responses. Platelet activation is accompanied by degranulation, resulting in the release of stromal cell-derived factor 1 alpha (SDF-1α) [55], which facilitates the recruitment of mast cells to the perivascular space, as well as by surface expression of P-selectin, enabling PNA formation. PNA formation subsequently triggers the extravasation and activation of neutrophils in the perivascular space of the expanding collaterals. Neutrophil-derived reactive oxygen species (ROS) in turn trigger mast cell degranulation [9]. Our Giemsa-stained tissue samples showed that Sildenafil treatment was associated with a significant increase in recruited mast cells, as well as enhanced mast cell degranulation on days 1 and 3 after FAL. Although no direct connection between Sildenafil and mast cell biology has been described yet, previous studies have linked PDE5 inhibition with inhibitory effects on histamine release, thereby protecting the endothelial barrier [56,57]. In the present study, we show for the first time an association between Sildenafil and ROS-dependent mast cell activation. Thus, we propose that the increase in mast cell recruitment is mediated by an enhanced release of SDF-1α from activated platelets, while the intensified mast cell activation is facilitated by increased PNA formation following Sildenafil treatment during arteriogenesis.

Mast cell-derived mediators are required for further leukocyte recruitment to the perivascular space of the growing collateral arteries [9,58]. Hence, we investigated the macrophage populations surrounding the collateral arteries after Sildenafil treatment by immunofluorescence staining. Here, a significant promotion of macrophage recruitment was observed upon Sildenafil treatment on days 3 and 7 after FAL when compared to controls. Additionally, treatment with Sildenafil pronounced the M2-like polarization of the perivascular macrophage population on day 7 after FAL, marking its anti-inflammatory and pro-regenerative impact. These data agree with a study that showed that Sildenafil inhibits the pro-inflammatory monocyte-to-macrophage polarization [59]. Furthermore, Sildenafil application reduces pro-inflammatory markers in patients with vasculogenic erectile dysfunction and facilitates regenerative processes in an in vivo wound healing model [60,61,62]. Similarly, during arteriogenesis, macrophages accumulate in the surroundings of the collateral arteries. Here, they enable extracellular matrix degradation and vascular cell proliferation, which are crucial for collateral artery growth [10,63]. Thus, we propose Sildenafil as a feasible drug to shift immune cell populations from pro-inflammatory to pro-regenerative phenotypes, a process that has already been described in previous studies [64,65,66].

Initially, Sildenafil was developed as a potential treatment for angina due to its vasodilating properties. During the first clinical trials, researchers noticed its effect of inducing penile erections. This unexpected finding redirected its development, leading to its approval for erectile dysfunction [67]. Today, the clinical use of Sildenafil has expanded from its original indication for erectile dysfunction to include treatment of PAH [23]. Additionally, recent studies have proposed the use of Sildenafil for arteriovenous fistula maturation [68], wound healing [61,62], and the prevention of in-stent restenosis [28]. The findings of the present study suggest a new potential field of application for Sildenafil in promoting natural bypass growth in patients with various CVDs, including peripheral and coronary artery disease.

Despite common adverse effects such as headache or flushing, Sildenafil is generally considered a safe and well-tolerated drug [69]. Its overall safety profile is supported by extensive clinical use across different indications [22,70,71]. However, factors such as interindividual differences, including polymorphisms in the *PDE5A* gene and those of other signaling molecules in the NO pathway, may influence treatment responses and should be considered when evaluating patient suitability [72,73]. For example, genetic variations in *PDE5A* or other downstream mediators of cGMP signaling could result in differential drug responses, affecting both the efficacy and safety of Sildenafil. These considerations further support a careful yet promising evaluation of Sildenafil as a positive modulator of arteriogenesis. The dose used in our study (10 mg/kg) reflects established murine models for effective PDE5 inhibition. While direct translation of this dose to humans is limited by species-specific pharmacokinetics [74], therapeutically effective doses for promoting arteriogenesis are expected to fall within clinically approved Sildenafil regimens. Nevertheless, future clinical studies are needed to define the optimal dosing for promoting natural bypass growth in patients.

In conclusion, the present study demonstrates that PDE5 inhibition by Sildenafil enhances blood flow recovery following femoral artery ligation by promoting the growth of collateral arteries. This effect is mediated by increased interactions between platelets and leukocytes, leading to a shift toward a regenerative form of sterile perivascular inflammation, which includes the accumulation of M2-like polarized macrophages, a process that Sildenafil further supports. Therefore, we propose that Sildenafil could be a clinically available drug to treat patients with cardiovascular occlusive diseases.

## 4. Materials and Methods

### 4.1. Animal Model and Drug Application

All animal procedures were conducted in compliance with the European Directive 2010/63/EU. Approval for all procedures involving animals was granted by the Bavarian Animal Care and Use Committee (Regierung von Oberbayern, Approval IDs ROB-55.2-2532.Vet_02-17-99, approval date 8 December 2017 and ROB-55.2-2532.Vet_02-22-99, approval date 29 March 2023). The experimental mice were maintained under well-controlled standard laboratory conditions, featuring a 12-h light and 12-h dark cycle to simulate natural environmental conditions. The mice had continuous access to a standard diet and drinking water. Enrichment was provided by coarse bedding, gnawing sticks, tunnels, and cellulose tissues.

In our study, healthy male SV129 wild-type mice, aged between 8 and 12 weeks and sourced from Charles River Laboratories (Sulzfeld, Germany) or bred in-house, were utilized. The mice were allocated to treatment groups using simple randomization based on cage assignment to minimize selection bias. Outcome assessments were performed unblinded due to practical constraints, with standardized protocols applied to reduce potential bias. No animals were excluded during the experimental period. The treatment involved either the administration of Sildenafil (10 mg/kg body weight, Hycultec, Beutelsbach, Germany), a PDE5-inhibitor, diluted in the drinking water, or a control treatment with standard laboratory drinking water, beginning one day before femoral artery ligation (FAL). The concentration of Sildenafil was selected based on a previous study [74]. For the dose calculation, the daily water consumption was assumed to be approximately 5 mL per mouse, based on prior observations, and was monitored throughout the experiment. To assess the proliferation of vascular cells following FAL, the experimental mice received daily intraperitoneal injections of 100 μL of bromodeoxyuridine (BrdU, 50 mg/kg/day) dissolved in phosphate-buffered saline (PBS, Merck, Darmstadt, Germany) starting immediately after FAL.

### 4.2. Femoral Artery Ligation (FAL)

The murine hindlimb model of arteriogenesis is a well-established experimental technique used to investigate the process of collateral artery growth [32]. In this model, to induce arteriogenesis, the superficial femoral artery on one hindlimb is carefully occluded using a surgical ligature, positioned just distal to the origin of the profunda femoris artery. The contralateral limb undergoes a sham surgery, where the artery is manipulated without actual ligation, serving as an internal control for comparative analysis.

To ensure the mice remained unconscious and pain-free throughout the procedure, a calibrated combination of anesthetics was administered. This included fentanyl at a dose of 0.05 mg/kg bodyweight (CuraMED Pharma, Karlsruhe, Germany), along with midazolam at 5 mg/kg bodyweight (Ratiopharm GmbH, Ulm, Germany), and medetomidine at 0.5 mg/kg bodyweight (Pfister Pharma, Berlin, Germany). For postoperative analgesia, the mice received a subcutaneous dose of buprenorphine (0.1 mg/kg body weight, Dechra Veterinary Products Deutschland GmbH, Aulendorf, Germany) immediately after FAL and on the first and second postoperative days.

### 4.3. Laser-Doppler Perfusion Imaging

To assess the blood flow recovery in the murine hindlimb after FAL, we conducted laser-Doppler imaging (LDI) using the Moor LDI system (Moor LDI 5061, Moor Software v3.01; Moor Instruments, Remagen, Germany). LDI is a precise, non-invasive method used to repeatedly evaluate blood flow recovery [75]. To ensure consistency and reduce variability due to environmental factors, the animals were housed in a thermostatically controlled chamber that maintained a stable temperature of 37 °C throughout the measurements. LDI was performed at multiple time points: before the femoral artery ligation procedure, immediately following the intervention, and again on postoperative days 3 and 7. For the quantification of blood flow recovery, a standardized grid comprising 45 measurement points was applied to each paw, as illustrated in Figure 1. The perfusion values recorded from the ligated limb were compared to those from the contralateral limb, which underwent the sham surgery.

### 4.4. Tissue Harvesting

At the conclusion of the experimental period, blood samples from the anesthetized experimental mice were collected by performing a cardiac puncture with heparinized syringes, and the mice were sacrificed through cervical dislocation. Following euthanasia, a specialized perfusion technique was employed to ensure preservation of the hindlimb vasculature. An aortic catheter was utilized to perfuse the hindlimb vascular system first with 20 mL of a vasodilating adenosine buffer, comprising 1% adenosine and 5% bovine serum albumin (BSA) dissolved in PBS (all reagents sourced from Sigma-Aldrich, St. Louis, MO, USA). This was immediately succeeded by the infusion of 20 mL of a fixative solution containing 3% paraformaldehyde (Merck, Darmstadt, Germany) in PBS to stabilize the tissue for subsequent analysis.

Once the vasculature was perfused, the adductor muscles were excised from the hindlimb and immersed in a 15% D(+) sucrose solution (AppliChem GmbH, Darmstadt, Germany) for one hour to enhance tissue preservation. The samples were then transferred to a 30% sucrose solution overnight to ensure complete infiltration. Finally, these prepared tissues were embedded in Tissue-Tek compound (Sakura Finetek Germany GmbH, Umkirch, Germany) for optimal preservation. The embedded samples were stored at −80 °C and subsequently sectioned into 10-μm cryosections, which were then prepared for detailed histological and immunofluorescence examination.

### 4.5. Immunofluorescence and Histological Staining

To visualize proliferating vascular cells in the growing collateral arteries and to examine surrounding immune cell populations, immunofluorescence staining was performed on 10-micrometer-thick cryosections of the adductor muscles containing the superficial growing collateral arteries. All stained samples were imaged using a Leica DM6 B epifluorescence microscope (Leica Microsystems, Wetzlar, Germany) equipped with a 40× objective, which allowed high-resolution visualization of the stained cells. The acquired images were subsequently processed and analyzed with Fiji (Image J, Version 1.54p), an open-source image analysis platform commonly used for quantitative evaluation of fluorescence microscopy data [76]. All images include a scale bar, the dimensions of which are specified in the corresponding figure legends.

To specifically identify proliferating cells, tissue sections from harvested adductor muscles were obtained on day 7 following the FAL procedure. These sections underwent DNA denaturation by treatment with 1 N hydrochloric acid (Merck, Darmstadt, Germany) for 30 min at 37 °C, followed by permeabilization of cellular membranes using a solution of 0.2% Triton X-100 (AppliChem, Darmstadt, Germany). After these preparations, the sections were blocked with 10% goat serum and incubated overnight with a rat anti-mouse BrdU antibody (Abcam, Cambridge, UK, ab6326, diluted to 1:50), which binds specifically to the incorporated BrdU, a synthetic thymidine analog, marking proliferating cells. Following the overnight incubation, the sections were rinsed with a wash buffer composed of 0.5% BSA and 0.1% Tween in PBS (all chemicals sourced from Merck, Darmstadt, Germany). Subsequently, a goat anti-rat Alexa Fluor 546-conjugated secondary antibody (Thermo Fisher Scientific, Waltham, MA, USA, A11081, diluted to 1:100) was applied to enable the visualization of the BrdU-labeled proliferating cells. Collateral diameters were calculated using this formula: Diameter=Circumference ×π.

To detect and distinguish macrophage subtypes within the perivascular area of the growing collateral arteries, adductor tissue sections were subjected to immunofluorescence staining. Macrophages were first labeled with an anti-CD68 (Abcam, Cambridge, UK, ab201844, 1:200) antibody, and the regenerative M2-like macrophage subtype was identified by an anti-mannose receptor c type 1 (MRC1)-antibody (Abcam, Cambridge, UK, ab64693, 1:200). This primary staining step was followed by employing an Alexa Fluor 546-conjugated anti-rabbit IgG secondary antibody (Thermo Fisher Scientific, Waltham, MA, USA, A10040, 1:200). To outline the collateral arteries, endothelial cells were visualized using an Alexa Fluor 647-conjugated anti-CD31 antibody (BioLegend, San Diego, CA, USA, 102516, diluted to 1:100) and smooth muscle cells were marked with an Alexa Fluor 488-conjugated anti-α-smooth muscle actin (ACTA2, Sigma-Aldrich, Darmstadt, Germany, F3777, diluted to 1:400). Nuclear counterstaining was performed with DAPI (Thermo Fisher Scientific, Waltham, MA, USA, 62248), diluted to 1:1000 from the provided 1 mg/mL solution.

For staining, we used negative controls lacking the primary antibody or the corresponding isotype antibody for prelabeled antibodies to evaluate background signal and nonspecific binding. Accordingly, BrdU staining was controlled using a rat IgG2a, κ isotype antibody (Abcam, Cambridge, UK, ab18450, diluted to 1:50). Staining for MRC1 was controlled using a rabbit IgG isotype antibody (Abcam, Cambridge, UK, ab37415, diluted to 1:200). CD68 and ACTA2 stains were controlled using a rat IgG2a, κ isotype antibody (BioLegend, San Diego, CA, USA, 400525), applied at dilutions of 1:200 and 1:400, respectively. CD31 staining was controlled using a rat IgG2a, κ isotype antibody (BioLegend, San Diego, CA, USA, 400526, diluted to 1:100). Non-specific binding of the secondary antibodies (Alexa Fluor 546-conjugated anti-rabbit IgG antibody from Thermo Fisher Scientific, Waltham, MA, USA, A10040 and anti-rat Alexa Fluor 546-conjugated antibody from Thermo Fisher Scientific, Waltham, MA, USA, A11081) was assessed by omitting the primary antibody.

Using Giemsa staining, we assessed mast cell recruitment and degranulation in adductor muscle tissue sections that were collected 24 h and 3 days after FAL, adhering to a standard protocol [77].

### 4.6. Flow-Cytometry

To characterize circulating immune cell populations and assess the extent of platelet–neutrophil aggregate and monocyte–platelet aggregate formation during arteriogenesis, we performed multicolor flow cytometry analyses. Whole blood was obtained 24 h after femoral artery ligation (FAL) by cardiac puncture from anaesthetized mice into heparinized syringes to prevent coagulation. Before staining, red blood cells were removed by incubating the samples with a diluted erythrocyte lysis buffer (1:10 dilution of the 10× BD Biosciences, Franklin Lakes, NJ, USA, lysing solution, 349202, prepared in distilled water).

Following lysis, the remaining leukocytes and platelets were subjected to surface marker staining using fluorophore-labeled antibodies to distinguish specific cell subsets. CD11b-PE (BioLegend, San Diego, CA, USA, 101208, 1:300) was used as a general marker for myeloid cells, while CD115-Brilliant Violet 421 (BioLegend, San Diego, CA, USA, 135513, 1:300) enabled circulating monocyte detection and Ly-6G/GR-1-APC (BioLegend, San Diego, CA, USA, 108412, 1:400) neutrophil identification. CD41-FITC (BioLegend, San Diego, CA, USA, 133903, 1:400) was used for platelet labeling. Additionally, a fixable viability dye (Invitrogen, Waltham, MA, USA, EF780, 1:1000) was incorporated into the staining panel to discriminate between live and dead cells, thereby increasing the reliability of downstream quantification.

To control for non-specific staining in flow cytometric analyses, fluorochrome-conjugated isotype control antibodies were included. CD11b-PE staining was controlled using a PE-conjugated rat IgG2b, κ isotype control antibody (BioLegend, San Diego, CA, USA, 400608, diluted to 1:300). CD115-BV421 staining was controlled using a Brilliant Violet 421-conjugated rat IgG2a, κ isotype control antibody (BioLegend, San Diego, CA, USA, 400549, diluted to 1:300). Ly-6G/GR-1-APC staining was controlled using an APC-conjugated rat IgG2b, κ isotype control antibody (BioLegend, San Diego, CA, USA, 400612, diluted to 1:400). CD41-FITC staining was controlled using a FITC-conjugated rat IgG1, κ isotype control antibody (BioLegend, San Diego, CA, USA, 400406, diluted to 1:400).

Data acquisition took place on a BD LSRFortessa™ flow cytometer (Becton, Dickinson and Company, Franklin Lakes, NJ, USA). The analyses were performed by employing FlowJo (Version 10, Becton, Dickinson and Company, Franklin Lakes, NJ, USA). Single-cell suspensions were first gated for singlets, followed by exclusion of dead cells. From the viable singlet population, CD11b-positive cells were selected. Within this CD11b-positive population, neutrophils were identified as GR-1hi/CD115lo and monocytes as GR-1lo/CD115hi. Platelet–neutrophil aggregates (PNA) were defined as cells double-positive for CD41 and GR-1 within the neutrophil gate, whereas platelet–monocyte aggregates (MPA) were identified as cells double-positive for CD41 and CD115 within the monocyte gate.

### 4.7. Differential Blood Analysis

To investigate the distinct blood cell subtypes present in animals subjected to femoral artery ligation, we performed a comprehensive differential blood analysis utilizing the ProCyte DX device (Idexx, Westbrook, ME, USA), which was specifically configured for mouse blood samples. Blood samples were obtained through cardiac puncture from mice that had been appropriately anesthetized as described above. This procedure took place 24 h post-FAL, ensuring we captured relevant changes in the blood profile due to the surgical intervention or the treatment with Sildenafil. The blood collection was conducted immediately prior to neck dissection.

### 4.8. Statistical Analyses

Statistical analyses were carried out using GraphPad Prism 10 (GraphPad Software, La Jolla, CA, USA, Version 10.6.1). The findings are shown as mean values, along with the standard error of the mean, to illustrate the variability associated with the group mean and to enable easy comparison between the groups. A *p*-value under 0.05 was considered statistically significant. The normality of the data was assessed prior to further statistical analyses using the Shapiro–Wilk test. Each figure legend includes the specific statistical tests applied to the respective datasets. The exact *p*-values are reported consistently in all figures.

To determine the necessary sample sizes, we utilized G*Power software (Version 3.1.9.2). The calculation occurred before the study began and was based on expected effect sizes derived from preliminary data and previous research using the same animal model.

## Data Availability

The original data presented in this study are included in the article. Further inquiries can be directed to Lisa Bobrowski (lisa.bobrowski@med.uni-muenchen.de).

## Abbreviations

The following abbreviations are used in this manuscript:

ACTA2	Anti-alpha smooth muscle actin
ADP	Adenosine diphosphate
BrdU	Bromodeoxyuridine
BSA	Bovine serum albumin
CVDs	Cardiovascular diseases
FAL	Femoral artery ligation
GPIb	Glycoprotein Ib receptor
MPA	Monocyte–platelet aggregate
MRC1	Mannose receptor C type 1
PAH	pulmonary arterial hypertension
PAR1	protease-activated receptor 1
PBS	Phosphate-buffered saline
PDE5	Phosphodiesterase-5
PKG	Protein kinase G
PLA	Platelet–leukocyte aggregate
PNA	Platelet–neutrophil aggregate
ROS	Reactive oxygen species
SMC	Smooth muscle cell
VWF	Von Willebrand factor
